# Alternative Lengthening of Telomeres (ALT) in Tumors and Pluripotent Stem Cells

**DOI:** 10.3390/genes10121030

**Published:** 2019-12-10

**Authors:** Shuang Zhao, Feng Wang, Lin Liu

**Affiliations:** 1 College of Life Sciences, Nankai University, Tianjin 300071, China; 15002266961@163.com; 2 State Key Laboratory of Medicinal Chemical Biology, Nankai University, Tianjin 300071, China; 3 Department of Genetics, School of Basic Medical Sciences, Tianjin Medical University, Tianjin 300070, China; wangf@tmu.edu.cn

**Keywords:** alternative lengthening of telomeres, telomerase, DNA damage, pluripotent stem cells, telomere maintenance mechanism, genome stability

## Abstract

A telomere consists of repeated DNA sequences (TTAGGG)n as part of a nucleoprotein structure at the end of the linear chromosome, and their progressive shortening induces DNA damage response (DDR) that triggers cellular senescence. The telomere can be maintained by telomerase activity (TA) in the majority of cancer cells (particularly cancer stem cells) and pluripotent stem cells (PSCs), which exhibit unlimited self-proliferation. However, some cells, such as telomerase-deficient cancer cells, can add telomeric repeats by an alternative lengthening of the telomeres (ALT) pathway, showing telomere length heterogeneity. In this review, we focus on the mechanisms of the ALT pathway and potential clinical implications. We also discuss the characteristics of telomeres in PSCs, thereby shedding light on the therapeutic significance of telomere length regulation in age-related diseases and regenerative medicine.

## 1. Introduction

Telomeres consist of tandem TTAGGG repeats, ending with an approximate 50–500 nt G-rich 3’-strand overhang [1,2]. Telomeres are enclosed by the shelterin protein complex, which contains the double-strand telomeric DNA binding factors TRF1 and TRF2, the TRF2-interacting protein RAP1, the bridging factor TIN2, and the telomeric ssDNA-binding protein POT1 and its direct interactor TPP1 [3]. Shelterin proteins bind to the telomere structure and mediate the formation of a telomeric loop (T-loop) in which the single-strand 3’ overhang is concealed into a D-loop structure [4,5]. In somatic cells, telomeres continuously shorten with cell division, resulting in an accumulation of DNA damage that triggers cellular senescence [6,7,8]. In stem cells and about 85% of cancer cells, the telomeres could be elongated by telomerase through adding TTAGGG repeats to the chromosome ends. However, in the other 15% of cancer cells, telomeres are maintained by an alternative lengthening of telomeres (ALT) mechanism, which mainly relies on homologous recombination (HR) between sister chromatids. This review focuses on the mechanisms of ALT in tumors and pluripotent stem cells as well as the implications for related potential therapies.

## 2. Telomere Function in Maintaining Genomic Stability 

During lagging strand synthesis, the 5’ end cannot be replicated entirely due to the end replication problem, resulting in gradual loss of chromosomal end at each round of cell division [9]. Telomeres located at chromosome ends serve as an extendable DNA structure to solve this problem. Moreover, telomeres are recognized as difficult-to-replicate sites which are sensitive to replication stress and additional replication factors, including helicase, DNase, topoisomerase, and other DNA binding proteins that are required to properly replicate the telomeric dsDNA [10]. The mutation of these proteins induces telomere dysfunction or damage, and eventually leads to premature aging [11,12,13]. The telomere binding protein deficiency leads to “telomere uncapping” and eventually chromosomal instability and cell death. For example, the loss of telomeric dsDNA-binding protein TRF2 would cause ATM-dependent chromosomal fusion by NHEJ (Non-homologous end joining) [14]. In addition, the depletion of POT1 leads to aberrant RPA (replication protein A) accumulation and homologous recombination at telomeres, and consequently genomic instability and replicative senescence [15]. Both telomere shortening and telomere deprotection significantly increase the risk of tumorigenesis, especially when the tumor suppressor gene p53 is ablated. Consistently, mice with hyper-long telomere live longer and have less spontaneous tumor incidence [16].

Other telomere accessory factors and telomeric structures have also been identified to play essential roles in telomere integrity. For instance, telomere-repeat-encoding RNA (also referred to as TERRA) transcribed from the telomeric DNA, has been shown as a potential component of eukaryotic telomeres and to play essential roles in telomere homeostasis. TERRA transcription is repressed upon telomere elongation, mediated by the increased HP1 α and trimethylated H3K9 density [17]. Abnormal heterochromatin status induces TERRA expression deficiency and augmentation of telomere dysfunction-induced foci (TIF) [18]. Telomere 3’-strand overhang is thought to invade double-stranded telomeric DNA, resulting in a bulging duplex lariat construction known as T-loop that masks the 3’ end of the telomere from being recognized as a single strand or double strand DNA break. Thus, the generation of G-tail and T-loop is essential for telomere length and integrity maintenance [19,20]. G-quadruplex is another structure arisen from the self-stacking of two or more guanine quartets, frequently observed at the telomere region [21,22]. Also, loss-of-function mutations in DNA helicases, which have G-quadruplex unwinding activity link to telomere maintenance deficiency and genome instability [23,24,25].

## 3. Telomere Maintenance by Telomerase or ALT

To counteract the telomere loss and bypass replicative senescence, cells must establish a telomere maintenance mechanism (TMM), which permits prolonged proliferative potential [26,27,28]. The vast majority of the cancer cells and stem cells use the telomerase to lengthen telomeres [7]. Extension of telomeres by the telomerase catalytic process consists of several sequential stages. Telomerase is recruited to telomeric DNA via interaction with TPP1 OB-fold then taken to the 3’ terminal end via paring of the alignment fragment of TERC (telomerase RNA template) to the telomere [29,30,31]. Subsequently, the G-strand is reserve-transcribed by TERT (telomerase reverse transcriptase) using TERC as a template. The newly synthesized long G-overhang is covered by the CST complex, which displaces telomerase and recruits primase-Pol α to fill in the C-strand and produce the entire double strand telomeric DNA [32,33]. 

Approximately 10–15% of tumor cells elongate their telomeres using a recombination-based alternative lengthening mechanism. Interestingly, the correlation between alternative lengthening of telomeres (ALT) and prognosis varies among different cancer types and patients. In soft tissue sarcomas, ALT is associated with lower survival than telomerase activation [34]. In osteosarcomas, there is no difference in the clinical outcome [35]. However, ALT is associated with a better patient outcome in glioblastoma [36]. Differential prognosis of ALT may rely on the different genetic and epigenetic events responsible for TMMs. The preference to use one TMM rather than the other varies among tumor types [37]. Cancers with mesenchymal origin are more likely to use ALT, while cancers with an epithelial origin are more likely to activate telomerase [38,39]. However, the underlying reasons remain poorly understood. The bladder cancer cells epithelial-to-mesenchymal transition (EMT), which prevents cells from undergoing senescence during tumor development, promotes telomerase-to-ALT-like conversion, suggesting the factors involved in EMT may play an essential role in telomerase activity repression [40]. Moreover, telomerase activity is inhibited upon hybridization with ALT cells, indicating the existence of telomerase repressors in ALT-positive cells [41]. Additionally, telomerase positive tumors exhibit ALT hallmark upon anti-telomerase therapies [42,43]. Thus, ALT is considered as an intrinsic mechanism that coexists with telomerase as a back-up TMM during evolution in mammals [44]. Combined inhibition of telomerase and ALT may be needed to achieve extensive clinical efficacy.

Telomeric chromatin accessibility is thought to be essential for telomerase/ALT determination. Loss of telomeric heterochromatin markers, including H3K9me3 and H4K20me3, might induce ALT by promoting chromatin decondensation [45]. Additionally, loss of H3.3-specific chaperone ATRX or its cofactor DAXX progressively induces ALT activation via regulating the level of DNA methylation and heterochromatin [46]. During this process, heterochromatin protein HP1 may serve as a protein scaffold for ATRX recruitment [46,47]. Moreover, depletion of ATRX induced telomeric replication stress and DNA damage response [48]. Thus, ALT activation may be an adaptive response to ATRX depletion-induced telomere replication dysfunction [49]. 

## 4. Characteristics and Diagnosis of ALT

ALT in cancers is characterized by heterogeneous telomere sizes, which vary from extremely short (<1 kb) to abnormally long (>20 kb) in human cells [50,51]. Moreover, ALT cells are also featured with the presence of ALT-associated PML bodies (APBs), which are specialized promyelocytic leukemia (PML) protein bodies containing telomeric DNA, shelterin complex, and proteins related to DNA recombination (for example MRN, BLM, and WRN) [52,53,54,55]. Additionally, the appearance of extrachromosomal telomeric circular DNA, which may be the products of telomere trimming or self-replicating templates for telomeric DNA lengthening, is also assumed to be an exclusive feature of ALT-positive cell lines and tumors [56,57]. Telomeric sister chromatin exchange and abnormal insertion at chromosome ends are frequently observed in ALT-positive cells and considered a hallmark of ALT as well [58,59]. Lastly, telomere clustering, which offers a new platform for telomere recombination, was also known as a unique feature of ALT [60]. 

Given the above features, several approaches were developed to investigate ALT in tumors and cell lines. Telomere Restriction Fragment (TRF) southern blotting was regularly used to detect the heterogeneous telomere [61]. In line with the evidence that PML bodies localize with telomere DNA, the ALT-associated PML bodies (APBs) were used for ALT determination [35,62]. Bromodeoxyuridine (BrdU) labeling of telomeres in G2-phase cells and the examination of mitotic DNA synthesis (MiDAS) at telomeres, allowing the visualization of telomere DNA synthesis outside of S phase, is another straightforward assay to distinguish the ALT formation [60]. Additionally, the C-circle (CC) assay is a widely applied approach to detect ALT via rolling circle amplification for the extrachromosomal telomeres. The level of ALT activation can be quantified through the dot-blot analysis using 32P-labeled telomeric probes [56]. Moreover, the CC assay can detect the number of C-circles within just 30 ng of DNA, which allows the broad usage for the prevalence and prognostic analysis in ALT tumor samples [63]. 

## 5. How Is ALT Activated?

It has been widely accepted that DSBs at chromosomal ends trigger BIR (break-induced replication) -mediated telomeric DNA synthesis. Due to the extensive homology at telomere ends, a break-induced replication process is initiated where the broken end invades to the donor telomere then serves as a primer for initiation of DNA replication, relying on POLD3/4 [64]. Moreover, the replication stress at telomeres is also assumed to prime ALT as well, and the replication stress response protein SMARCAL1 was reported to associate with telomeres to inhibit the repair of DSBs and ensure ALT telomere maintenance, demonstrating that resolution of replication stress is a crucial step in the ALT mechanism [65,66]. In the absence of telomerase, telomere shortening leads to ssDNA accumulation at the telomere region, and HR (homologous recombination) machinery counteracts the ssDNA and elongates the telomere through the DNA damage response pathway [67]. In addition, the telomere length maintaining mechanism of telomerase-negative cells requires genes encoding HR-related proteins (including MRN complex, Rad51, Rad52, FANC proteins), suggesting HR are essential for conservative replication of telomeres, besides their DNA repair functions [68]. There are three routes of telomeres being HR-sorted by this type of telomeric DNA exchange: Equivalent telomeric sister chromatin exchange (T-SCE), inequivalent T-SCE, and non-sister chromatid exchange (No-SCE) [69]. Inequivalent T-SCE mainly leads to telomere length heterogeneity without a net gain of telomere length, whereas No-SCE, which may be induced by interchromatin HR and break-induced replication, results in increased telomere length [69].

Two distinct pathways are identified during ALT elevation [70]. One pathway requires RAD52, which binds DNA and facilitates the annealing process between complementary ssDNA strands [71,72]. Recruitment of RAD52 to the telomere is an SLX4-dependent process [73]. Furthermore, the replication intermediates generated in the absence of RAD52 can be processed by SLX4. Simultaneous deletion of *RAD52* and *SLX4* results in deficiency in mitotic fidelity and telomere dysfunctions, suggesting the accumulation of unresolved stalled forks and recombination intermediates, which may serve as barriers to DNA synthesis and lead to gradual telomere shortening [71]. The other ALT mechanism is independent of RAD52 but requires BLM and POLD3/4, suggesting that activation of this ALT pathway is mediated by a BIR-related process [64,70]. Nevertheless, both processes take place within APBs, which offer a “recombinogenic microenvironment” to facilitate ALT, and these two different repair syntheses rely on the nature of telomere lesions and cell cycle phases [70,71]. 

**Figure 1:**
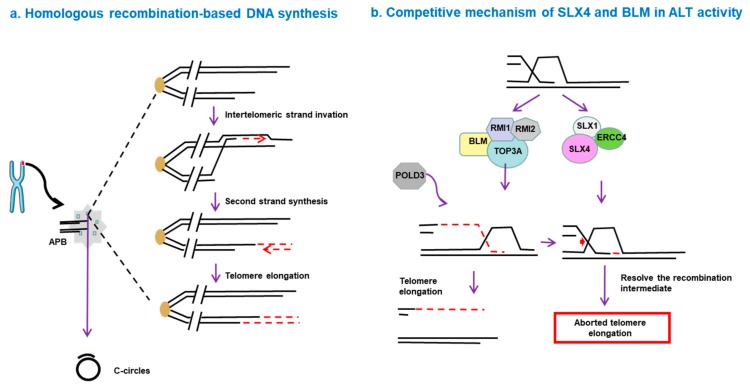
Homologous recombination-based telomere DNA synthesis. (**a**) DNA double-strand breaks can trigger telomere synthesis. A break-induced replication process is initiated when the broken end invades a donor telomere, followed by replication of the donor DNA sequence and invading DNA, resulting in increased telomere length. (**b**) Competitive mechanism of SLX4 and BLM in alternative lengthening of telomeres (ALT) activity. The BLM-TOP3A-RMI (BTR) complex is essential for ALT-mediated telomere synthesis. In this process, recombination intermediates can initiate POLD3-dependent telomere synthesis, followed by dissolution, without inducing telomere sister-chromatid exchange (T-SCE). However, this process is inhibited by the SLX4-SLX1-ERCC4 complex, which promotes the resolution of the recombination intermediates and leads to telomere exchange without telomere elongation.

BIR functions via an RFC–PCNA–Pol δ axis, independent of other canonical replisome components such as ATM, ATR and Rad51 [74]. Additionally, BLM-TOP3A-RMI (BTR) complex is necessary for ALT-mediated telomere synthesis. In this process, recombination intermediates can initiate large-scale POLD3-dependent telomere synthesis, followed by dissolution, without inducing T-SCE. However, this process is inhibited by the SLX4-SLX1-ERCC4 complex, which promotes the resolution of recombination intermediates, leading to telomere exchange without telomere extension [75] (Figure 1). The complexity of the ALT mechanism leads to different behaviors of ALT tumors in terms of disease progression and prognosis. Hence, a deep understanding of the molecular mechanisms of ALT pathways seems to be essential for diagnosis of ALT and discovery of novel drugs targeting this pathway.

## 6. ALT in Pluripotent Stem Cells (PSCs)

**Figure 2:**
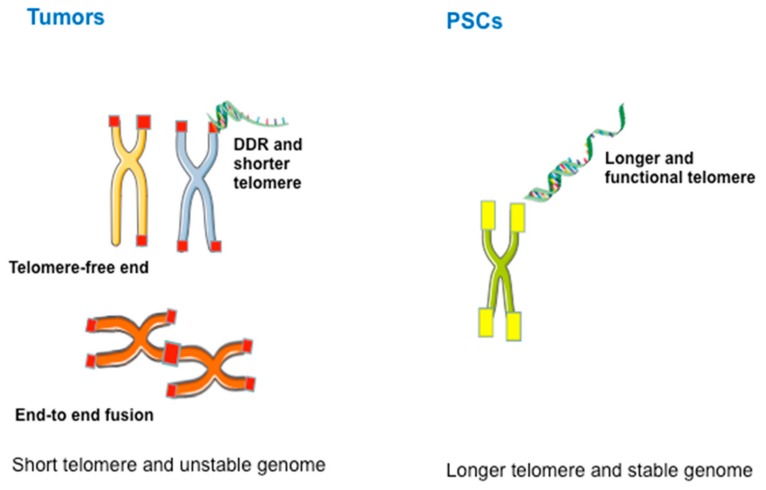
Distinctions between telomeres in tumor cells and pluripotent stem cells (PSCs). The genome of tumors is unstable and is characterized by heterogeneous telomeres, extrachromosomal DNA circles, ALT-associated promyelocytic leukemia (PML) bodies (APBs), and frequent T-SCEs, whereas PSCs exhibit longer functional telomeres and stable genomes.

Telomere maintenance is critical for the unlimited self-renewal, stemness, and genomic homeostasis of PSCs [76]. Telomere length represents another important criterion for defining stem cell pluripotency, and modulation of telomere length may present great potential in the application of PSCs in regenerative medicine [77]. Sufficient telomere length is also a requirement for the functionality of adult stem cells [78]. PSCs commonly express telomerase to maintain telomeres, and increasing evidence shows that the ALT-like pathway also plays a crucial role in telomere maintenance [77,79]. Both cancer cells (especially cancer stem cells) and PSCs rely on telomere maintenance for cell proliferation. However, telomeres and their length regulation show apparent differences between these two cell types. For example, the genomes of tumors with ALT are unstable, exhibiting heterogeneous telomeres, extrachromosomal DNA circles, APBs, frequent T-SCE, and dysfunctional telomeres. In contrast, PSCs maintain longer telomeres and stable genomes (Figure 2). The underlying mechanism remains unclear, but ALT in PSCs is mainly triggered by changes in epigenetic reprogramming [79], which provides an “open” chromatin state for activating ALT, rather than the harmful mutations that frequently occur in cancer cells. Additionally, ALT in cancer cells, but not in PSCs, involves mechanisms that negatively regulate telomere length by trimming telomeric DNA, resulting in the formation of t-circles [80].

Telomere length homeostasis is crucial for the genomic integrity of embryonic stem cells (ESCs) and must be maintained to prevent excessive telomere elongation. ESCs cultured under standard conditions in the presence of leukemia inhibitory factor (LIF) as well as feeders, can shuttle back and forth from a state that resembles a two-cell embryo-like state [81]. *Zscan4*, a two-cell embryonic gene expressed during zygotic genome activation, is essential for telomere extension in mESCs by T-SCE-dependent HR [82]. Expression of *Zscan4* is activated upon telomere shortening and reaches to the maximum level at the G2 phase of the cell cycle, which may represent a stage in which telomere extension can occur [83]. It is likely that two-cell genes, including *Zscan4*, are controlled by several regulators to maintain their appropriate activation in a small fraction of ESC populations, leading to efficient elongation of telomeres, followed by a telomerase-dependent mechanism for telomere maintenance [84]. Rif1, a telomere-associated protein, was initially identified in budding yeast that negatively regulates telomere length and plays an essential role in yeast telomere length homeostasis [85]. For differentiated mammalian cells, Rif1 was identified to play roles in DNA damage response and replication timing control but presents no functions related to telomeres [86,87]. In mouse ESCs, Rif1 regulates expression of *Zscan4* and other 2-cell genes by maintaining heterochromatic H3K9me3 histone methylation levels at subtelomeric regions. Thus, Rif1 acts as an essential factor for telomere length homeostasis by negatively regulating 2-cell genes [84]. Depletion of *Rif1* results in heterogeneous telomere elongation and shortening, similar to what is observed in ALT cancer cells [84].

**Figure 3:**
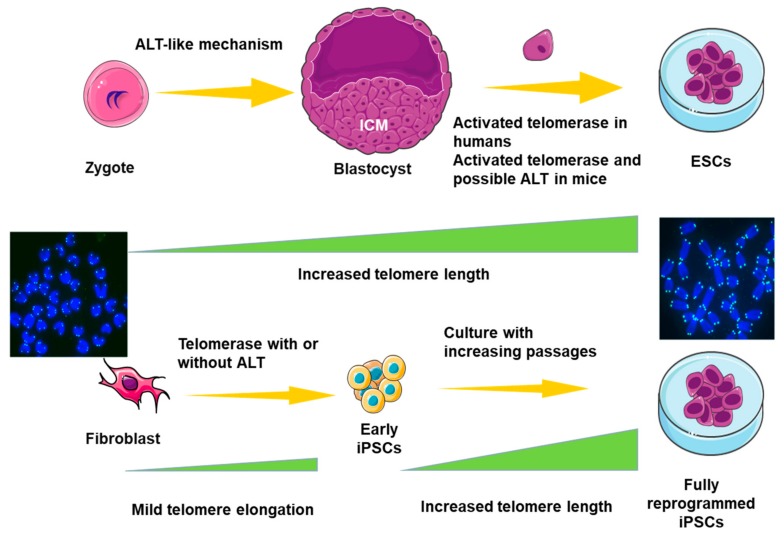
Telomere extension mechanism during acquisition of pluripotency. Telomeres of embryonic stem cells (ESCs) are extended during the early stage of derivation and reach a relatively stable level. The ALT mechanism is not observed in human ESCs, unlike the situation in mouse ESCs. Similarly, telomeres of iPSCs (induced pluripotent stem cells) are remodeled to a state resembling ESCs mainly by telomerase-dependent and possible ALT-like pathways and continuously elongate during the acquisition of fully reprogrammed iPSCs.

Telomeres of human ESCs are extended by telomerase during the early stage of derivation and reach a relatively stable level [88]. Interestingly, unlike human ESCs, many ALT features are also detected in telomerase-negative mouse ESCs with short telomeres [89]. Telomeres are extended by a mechanism resembling ALT-like activity during early embryo cleavage stage [90]. Despite telomere elongation during cleavage stages, the inner cell mass (ICM) of blastocysts still presents shorter telomeres compared to ESCs, and ESCs derived from the ICM in vitro exhibit elongation of telomeres to a relatively stable level depending on both telomerase [91] and an ALT-like mechanism [77] (Figure 3). This difference may be illustrated by the fact that ALT can emerge in naïve mouse ESCs but not in human ESCs, which more closely resemble the primed state of ESCs or epiblast stem cells in mice. Nevertheless, this assumption requires further evidence [79]. 

During the process of iPSC reprogramming, telomerase activity increases gradually before the emergence of endogenous pluripotency genes [92]. Ectopic expression of factors that could protect telomeres by increasing ALT-like activity, together with *Zscan4*, can increase the efficiency and pluripotency of iPSCs dramatically [93]. Additionally, small molecules that promote *Zscan4* expression and telomeric elongation by ALT-like pathway facilitate the generation of high-quality iPSCs [94,95]. Telomeres of iPSCs are remodeled to a state resembling ESCs mainly by telomerase-dependent and possible ALT-like pathways [92,96] and continuously elongate during the acquisition of fully reprogrammed iPSCs [95].

PSCs exhibit a more open telomeric chromatin structure, which becomes condensed during differentiation [97]. Hence, the telomeric structure of PSCs may be in a dynamic state that experiences remodeling in the process of differentiation [98]. The epigenetic state appears to play a critical role in the telomeric homeostasis of PSCs by affecting the process of ALT. Downregulation of H4K20me3 and H3K9me3 results in abnormally elongated telomeres [99,100]. Tet enzymes regulate telomere length by participating in epigenetic modification, with an essential role in pluripotency [101,102]. Moreover, ESC culture conditions can impact epigenetic modification and transcription and ultimately affect the telomere regulation mode of PSCs. For example, the 2i (MEK inhibitor and Gsk3β inhibitor)+LIF culture medium can decrease DNA methylation and H3K9me3 levels to affect telomeric homeostasis [81]. Additionally, DNA methylation and H3K9me3 prevent reprogramming in association with the negative regulation of telomere length. In contrast, DNA demethylation and increased histone acetylation can promote epigenetic reprogramming by lengthening telomeres [79]. Notably, PSCs derived from *Terc*-/- somatic cell nuclear transfer (SCNT) exhibit longer telomeres, higher pluripotency, and a greater self-renewal capacity than *Terc*-/- iPSCs, indicating that a telomerase-independent telomere elongation mechanism, possibly ALT, is more efficient in SCNT [103]. Hence, PSCs, including ESCs and iPSCs, must extend their telomeres during derivation and passaging to maintain their self-renewal ability and pluripotency [96]. A better understanding of how PSCs maintain their telomeres via telomerase and/or ALT-like activity is of significance in the fields of aging, stem cell biology, and regenerative medicine.

## 7. Potential Therapeutic Strategy Targeting ALT

Targeting of telomeres has received much attention regarding its potential clinical anti-cancer applications. At the molecular level, most of the cancer cells require one or the other TMM to maintain their telomeres to achieve immortalization [39,104]. Here, we list a number of potential therapeutic strategies in the treatment of tumors that rely on ALT.

Mutations in ATRX/DAXX as well as histone H3.3 are major causes of ALT activation in pediatric glioblastomas, pancreatic neuroendocrine cancers, and other cancers of the central nervous system [105,106,107]. Thus, it is likely that inactivation of ATRX/DAXX/H3.3 is an essential step in the generation of ALT in those tumors [108], which provides potential targets for anti-cancer therapies with transgenic technology. Recently, a screened mutant herpes simplex virus is shown to kill ATRX-deficient ALT tumors directly, through the recognition of ATRX [109]. Targeting ATRX/DAXX and telomerase simultaneously or separately may effectively induce tumor senescence and apoptosis in malignant gliomas. 

Several drugs targeting the cell cycle and DNA damage response have entered clinical trials for ALT tumors. Cell cycle G2/M checkpoint deficiency offers a potential vulnerability specific to ALT-positive cells. Thus, G2/M checkpoint depressors have been developed for ALT-positive cancers [110]. Additionally, non-S-phase telomere synthesis is considered as a valuable therapeutic target in ALT tumors due to its difference from canonical S-phase replication [60]. 

Roles of apoptosis in anti-tumor effects have been investigated in many different cancers, such as cervical cancer and Epstein-Barr virus (EBV)-positive B lymphocytes [111]. Suppression of telomere-related genes through telomerase and ALT mechanisms prevents development of laryngeal squamous cell carcinoma by facilitating cell apoptosis through a mechanism involving the PI3K/Akt pathway [112], which may provide a potential therapeutic strategy via promoting apoptosis. G-quadruplex ligands have been indicated to disturb telomere replication, with an anti-proliferative result [113,114]. Several G-quadruplex ligands with high affinity for telomeric G-quadruplexes are proved to have potential anti-tumor properties [115,116]. G-quadruplex stabilization might have synergistic effects with other DNA-damaging therapies, such as ionizing radiation, in ATRX-deficient tumors [117,118].

Most tumors use just one of the TMMs to maintain homeostasis. Nevertheless, the coexistence of ALT and telomerase has been identified both in vitro and in vivo [28,119]. Interestingly, osteosarcoma and neuroblastoma are characterized by inner-tumoral heterogeneity in TMM, where ALT and telomerase activity exist in different cells of the same tumor [120,121,122]. In contrast, breast cancer samples have revealed both telomerase and ALT within the same cell [123]. Moreover, after treatment with drugs targeting telomerase, a number of cancer cells escape death, switching from telomerase activity to ALT [104]. The molecular foundation underlying this switch or coexistence of both TMMs within the same cell or cell population remains to be elucidated. A characteristic description of these distinct cancers could improve our progression towards not only understanding the exact mechanisms of the two TMMs but also discovery of effective therapeutic strategies.

## 8. Perspectives

Based on the current understanding of the features of ALT, some of therapeutic strategies targeting this pathway have entered phase I/II clinical trials, such as the use of Trabectedin in treating soft tissue sarcoma [124]. Nevertheless, numerous puzzling problems remain regarding this specific mechanism: Why do differences in ALT or ALT-like activities exist in tumors, especially in cancer stem cells and PSCs? What are the potential differences in the mechanisms of ALT in these different cell types? Simultaneous single-cell measurements of telomeres and analyses of transcriptome, epigenome, or proteome in the same cell may resolve these issues in the future. Further studies are necessary to assess the unique biological characteristics and possible prognosis of ALT-positive cancers and will be significant in designing novel anti-cancer therapeutics targeting the ALT pathway.
